# Closed-loop Robots Driven by Short-Term Synaptic Plasticity: Emergent Explorative vs. Limit-Cycle Locomotion

**DOI:** 10.3389/fnbot.2016.00012

**Published:** 2016-10-18

**Authors:** Laura Martin, Bulcsú Sándor, Claudius Gros

**Affiliations:** Institute for Theoretical Physics, Goethe University FrankfurtFrankfurt am Main, Germany

**Keywords:** closed-loop robots, short-term synaptic plasticity, limit cycles, sensorimotor loop, self-organized locomotion, compliant robot

## Abstract

We examine the hypothesis, that short-term synaptic plasticity (STSP) may generate self-organized motor patterns. We simulated sphere-shaped autonomous robots, within the LPZRobots simulation package, containing three weights moving along orthogonal internal rods. The position of a weight is controlled by a single neuron receiving excitatory input from the sensor, measuring its actual position, and inhibitory inputs from the other two neurons. The inhibitory connections are transiently plastic, following physiologically inspired STSP-rules. We find that a wide palette of motion patterns are generated through the interaction of STSP, robot, and environment (closed-loop configuration), including various forward meandering and circular motions, together with chaotic trajectories. The observed locomotion is robust with respect to additional interactions with obstacles. In the chaotic phase the robot is seemingly engaged in actively exploring its environment. We believe that our results constitute a concept of proof that transient synaptic plasticity, as described by STSP, may potentially be important for the generation of motor commands and for the emergence of complex locomotion patterns, adapting seamlessly also to unexpected environmental feedback. We observe spontaneous and collision induced mode switchings, finding in addition, that locomotion may follow transiently limit cycles which are otherwise unstable. Regular locomotion corresponds to stable limit cycles in the sensorimotor loop, which may be characterized in turn by arbitrary angles of propagation. This degeneracy is, in our analysis, one of the drivings for the chaotic wandering observed for selected parameter settings, which is induced by the smooth diffusion of the angle of propagation.

## 1. Introduction

It has been argued (Pfeifer et al., 2007; Aguilar et al., 2016) that “robophysics,” defined as the pursuit of the discovery of biologically inspired principles of self generated motion, may constitute a promising road for eventually achieving life-like locomotor abilities. Distinct principles such as predictive information (Ay et al., 2008), surprise minimization (Friston, 2011), chaos control (Steingrube et al., 2010), empowerment (Salge et al., 2014), homeokinesis (Der and Martius, 2012), cheap design (Montúfar et al., 2015), and curiosity (Frank et al., 2014) have been studied in this context. Behavior, resulting from guided self organization (Prokopenko, 2009) or autonomous adaption (Chiel and Beer, 1997), may be generated in addition through suitable synaptic (Der and Martius, 2015; Der, 2016) and intrinsic (Sándor et al., 2015) plasticity rules.

Here we point out, that complex dynamics may be generated through a transient plasticity mechanism widely present in the brain. Short-term synaptic plasticity (STSP) (Fioravante and Regehr, 2011; Regehr, 2012) is an activity induced transient modulation of the synaptic efficiency, which may lead either to facilitating or to depressing behavior lasting from a few hundred to a few thousand milliseconds. STSP has been argued, besides others, to be relevant or causal for working memory (Barak and Tsodyks, 2014), for the facilitation of time sequences of alternating neural populations (Carrillo-Reid et al., 2015), for motor control in general (Nadim and Manor, 2000), and for the sculpting of rhythmic motor patterns (Jia and Parker, 2016) in particular. Plasticity mechanisms similar to STSP have also been shown to allow for stable gaits (Toutounji and Pasemann, 2014) in neural networks which are distinctively simpler than the ones used conventionally for bio-inspired controllers (Schilling et al., 2013).

**Figure 1:**
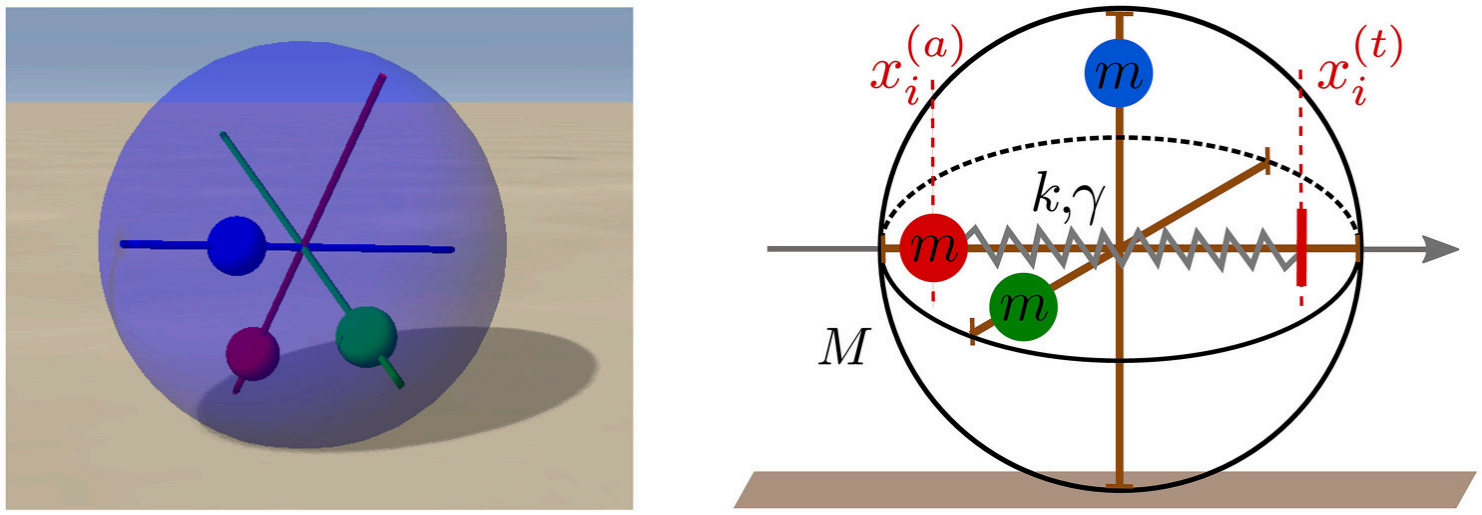
**Left: A snapshot of the spherical robot from the LPZRobots simulation environment (Martius et al., 2013)**. The three weights (red, green, and blue) can move along the respective rods without interference. **Right**: A sketch of the robot with the three perpendicular rods together with the three weights of mass *m*. The red vertical dashed lines show the actual position $x_{i}^{\left(a\right)}$ and a putative target position $x_{i}^{\left(t\right)}$ of the red weight along its rod. A damped spring with spring constant *k* and damping γ then pulls the weight toward the target position, which is given in turn by the output of a controlling neuron (compare Figure 2).

In this study we use the LPZRobots physics simulation package (Der and Martius, 2012) for the investigation of the spherical three-axis robot illustrated in Figure 1. This robot is driven exclusively by STSP, with locomotion coming to a stillstand both in the absence of synaptic plasticity and when the feedback from the environment is cut off, e.g., when the gravitational constant is set to zero. We find a surprisingly large palette of self-organized motion primitives, which includes a chaotic phase. The locomotion observed is flexible, in all modes, readjusting seamlessly to disturbances like the collision of the robot with obstacles.

The capability of STSP to have a large impact on locomotion can be traced back in our analysis to the destabilizing effect short-term synaptic plasticity may have on attracting states of the controlling network, inducing attractor-to-attractor transitions within timescales of the order of a few hundred milliseconds. We corroborate this findings by short-circuiting the sensori-motor loop, viz by taking out the environment. Transitions between distinct limit cycles within the full sensori-motor loop are found in addition in the chaotic mode.

## 2. Materials and methods

### 2.1. Tsodyks-markram model with full depletion

The way neurotransmitters are released through the synaptic cleft may change transiently upon repeated presynaptic activity (Tsodyks and Markram, 1997), both for excitatory (Wang et al., 2006) and for inhibitory (Gupta et al., 2000) synapses. Physiologically this is, on the one side due to an increase of the Ca-concentration *u* ∈ [1, *U*_*max*_] within the presynaptic bulge, facilitating the release of the respective neurotransmitter, and, on the other side, due to the decrease of the number φ ∈ [0, 1] of available vesicles of neurotransmitters. We use here with

(1) $$\begin{array}{l} \dot{u}=\frac{U(y)-u}{T_{u}},\;U(y)=1+(U_{max}-1)y\\ \dot{\varphi }=\frac{\Phi (u,y)-\varphi }{T_{\varphi }},\;\Phi (u,y)=1-\frac{uy}{U_{max}} \end{array}$$

a modified version of the original Tsodyks-Markram model (Tsodyks and Markram, 1997; Hennig, 2013), in which the the Ca-concentration *u* and the number of vesicles φ of a given synapse relax to target values *U* = *U*(*y*) and Φ = Φ(*u, y*), determined in turn by the level *y* ∈ [0, 1] of the presynaptic activity. A prolonged maximal presynaptic activity *y* ≡ 1 would lead with φ → 0 to a full depletion of the reservoir of vesicles.

The dynamics of the full depletion model (2.1) is determined by the relaxation time constants *T*_*u*_ and *T*_φ_, and by the maximal level *U*_*max*_ of the Ca concentration. For *U*_*max*_ = 1 a monotone depression is present, whereas *U*_*max*_ > 1 initially generates facilitation by a fast calcium influx, being annulled later on by the depletion of neurotransmitters. Overall, the synaptic efficiency is proportional to *uφ*, viz to the number of vesicles and to the release probability (which in turn is assumed to be proportional to *u*). We use *T*_*u*_ = 300ms and *T*_φ_ = 600ms, together with either *U*_*max*_ = 1 or *U*_*max*_ = 4. These values are within the typical range of what is physiologically observed (Gupta et al., 2000; Wang et al., 2006).

### 2.2. The robot

The movement of robot illustrated in Figure 1 is induced by the relative gravitational pull of the three weights, together with the rolling friction and angular momentum conservation. The individual neurons *i* = 1, 2, 3 are modeled as rate-encoding leaky integrators,

(2) $$\begin{array}{l} \;{\dot{x}}_{i}=-\Gamma x_{i}+\frac{w_{0}}{2pR}\left(x_{i}^{\left(a\right)}+pR\right)-z_{0}\sum_{j\neq i}u_{j}\varphi_{j}y(x_{j}),\\ y(x_{j})=\frac{1}{1+\exp(-ax_{j})}, \end{array}$$

where *x*_*i*_ and *y*(*x*_*i*_) are the respective membrane potentials and firing rates. Γ is the relaxation rate, *R* the diameter of the robot, *p* ∈ [0, 1] a rescaling factor, $x_{i}^{\left(a\right)}\in \left[-R,R\right]$ the sensory reading of the actual position of the weight on the rod, *w*_0_ > 0 the weight of excitatory input and *z*_0_ > 0 the magnitude of the inter-neural inhibitory connections. We note that the variables of the STSP, *u*_*j*_ and φ_*j*_, as described by Equation (2.1), depend only on the presynaptic activity and can hence be attributed altogether to the presynaptic neuron. For the slope of the sigmoidal *a* = 0.4 has been selected. The weight of the excitatory input *w*_0_ is not modulated here by short-term synaptic plasticity, corresponding to a direct sensory reading.

We selected with *p* = 1/2 a reduced range for the target position $x_{i}^{\left(t\right)}$,

(3) $$x_{i}^{\left(t\right)}=pR\left[2y(x_{i})-1\right],\;x_{i}^{\left(t\right)}\in [-pR,pR].$$

**Figure 2:**
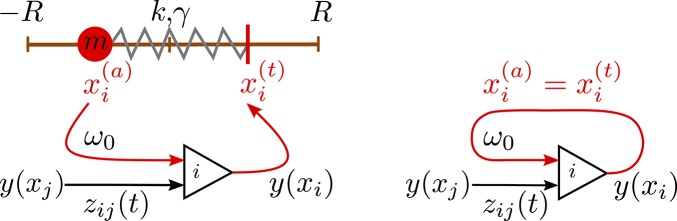
**Left: Sketch of the sensorimotor loop of the three-axis spherical robot illustrated in Figure 1**. The three weights *i* = 1, 2, 3 with masses *m* are each controlled by a single neuron. The excitatory input $w_{0}\left(x_{i}^{\left(a\right)}+pR\right)/\left(2pR\right)$ of neuron *i* is proportional to the proprio-sensory measurement of the actual position $x_{i}^{\left(a\right)}\in \left[-R,R\right]$ of the *i*-th mass (*p* ∈ [0, 1]). The neuron also receives inhibitory inputs −*z*_0_φ_*j*_*u*_*j*_*y*(*x*_*j*_) from the other two neurons (*j* ≠ *i*). The output *y*(*x*_*i*_) of the *i*-th neuron determines via $x_{i}^{\left(t\right)}=pR\left[2y\left(x_{i}\right)-1\right]$ the target position of the *i*-th mass. **Right**: A network of (three) neurons having the identical topology as the one of the three-axis spherical robot, but with the feedback of the environment short-cut by identifying the actual position $x_{i}^{\left(a\right)}$ with the target position $x_{i}^{\left(t\right)}$.

This choice allows to avoid dynamic overshooting of the weight when accelerated from its actual to the target position. The force accelerating the weight is calculated by the LPZRobots package by simulating a damped spring:

(4) $$m{\ddot{x}}_{i}^{\left(a\right)}\;=-k(x_{i}^{\left(a\right)}-x_{i}^{\left(t\right)})-\gamma \frac{d(x_{i}^{\left(a\right)}-x_{i}^{\left(t\right)})}{dt}+F_{i},\;x_{i}^{\left(a\right)}\to x_{i}^{\left(t\right)}\text{​​},$$

where *k* is the spring constant and γ the damping. Centrifugal and other induced forces, *F*_*i*_, act additionally in Equation (4) on the individual weights. The complete setup of the three-neuron network is illustrated in Figure 2.

### 2.3. Simulation parameters

The LPZRobots simulation environment (Der and Martius, 2012) is an interactive simulator based on the ODE (Open Dynamic Engine) (Smith, 2005). LPZRobots contains rigid body dynamics in terms of a library of basic primitive objects, such as spheres and cuboids, as well as a variety of joints, sensors and surface materials.

We used *roughness* = 0.8, *slip* = 0.01, *hardness* = 40 and *elasiticity* = 0.5 for the collision and friction properties together with *friction* = 0.3 (the rolling friction coefficient), *gravity* = −9.81 (the gravitational constant) and *noise* = 0 (for the global noise level). All parameters are in SI units. For the stepsize of the physical simulation *simstepsize* = 0.001 was used (corresponding to a millisecond). With *controlinterval* = 1 one ensures that the controller, viz Equation (2), is updated as often as the physics of the environment.

The robot itself has a diameter of 2*R* = 0.5, a mass off *M* = 1 and a *motorpowerfactor* = 120. The parameters for the damped oscillator (Equation 4) are *m* = 1, *k* = m^*^motorpowerfactor and $\gamma =2\sqrt{k*m}$ (critical damping). The relaxation rate for the membrane potential entering Equation (2) has been set to Γ = 20, retaining the bare excitatory and inhibitory weights, *w*_0_ and *z*_0_, as free simulation parameters.

## 3. Results

### 3.1. Emergent limit-cycle locomotion

**Figure 3:**
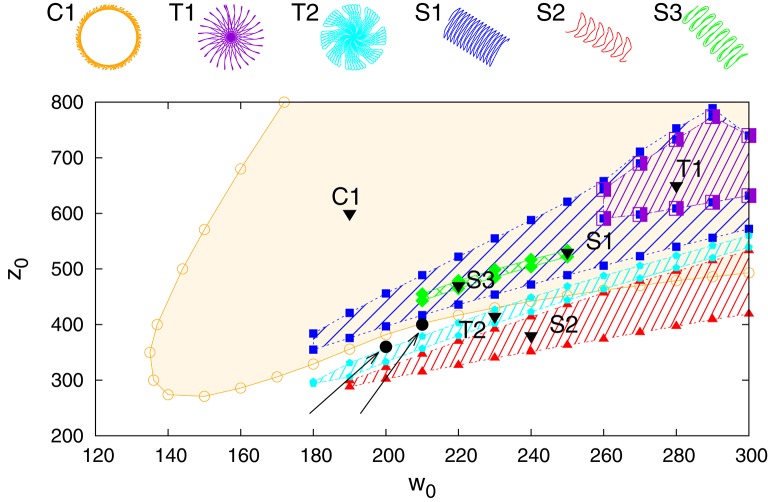
**Phase diagram for *U*_*max*_ = 1 in the parameter plane of excitatory (*w*_0_) and inhibitory (*z*_0_) synaptic weights**. On the top the different types of identified regular motion patterns are illustrated, tagged respectively with black triangles in the respective regions of stability (shaded areas). Close-up trajectories are given in Figure 4; for a comparison see also Supplementary Video 1. Examples of two parameter settings, (200, 360) and (210, 400), for which chaotic behavior is observed are indicated by black filled circles (at the tip of the respective arrows).

**Figure 4:**
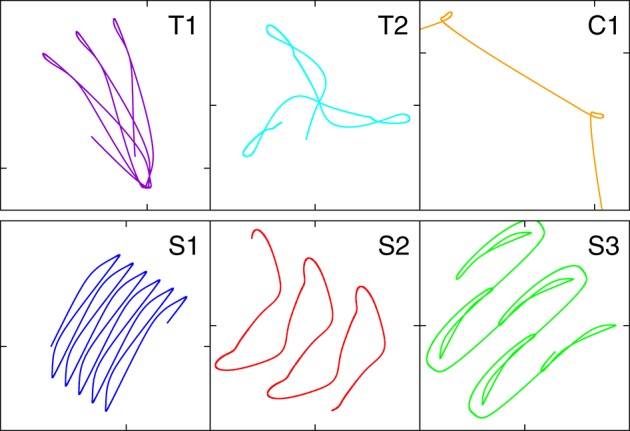
**A close-up of the trajectories in the plane of locomotion, for the parameters (*w*_0_, *z*_0_) tagged as black triangles in the phase diagram presented in Figure 3**. T1: (280, 650), T2: (230, 415), C1: (190, 600), S1: (250, 530), S2: (240, 380), S3: (220, 470).

In Figure 3 we present the stability regions for the various regular movement patterns found, with respective close-ups given in Figure 4. The results are for *U*_*max*_ = 1 (depressing short-term synaptic plasticity without Ca dynamics) and for the parameters specified in Section 2.3. They are obtained by adiabatically continuing stable states along a grid until stability is lost. Without STSP only a globally attracting fixpoint corresponding to a motionless robot is present. We note that regular motion arises for a wide range of bare excitatory (*w*_0_) and inhibitory (*z*_0_) synaptic weights. *z*_0_ needs however to be larger than *w*_0_.

All motion patterns observed are self-organized. There is no objective function (Gros, 2014), such as a maximal velocity, to be optimized. This implies that the quantitative features of the individual motion patterns change smoothly within their respective stability regions, and that one can identify the observed regular movement patters as stable limit cycles in the sensorimotor loop (Sándor et al., 2015). Fast switching between motion primitives would be possible by a putative overarching controller, since more than one limit cycle may be stable for given synaptic weights *w*_0_ and *z*_0_. Interactions between robots or with external obstacles might also lead to the automatic selection of another coexisting mode (see for instance Supplementary Video 1).

**Figure 5:**
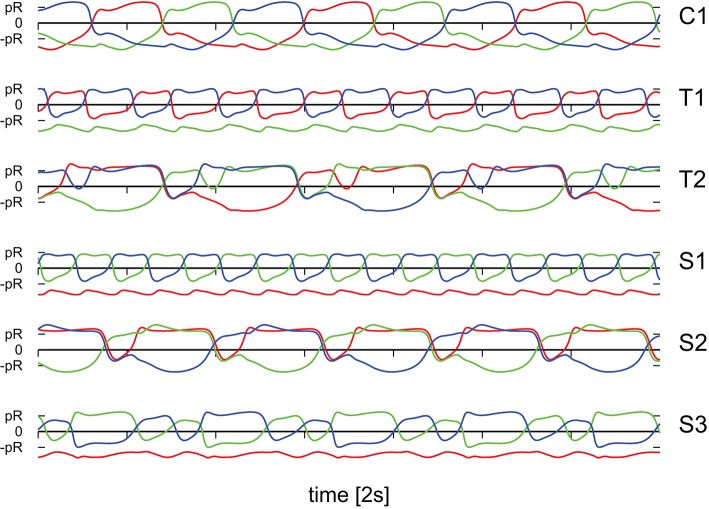
**The positions $x_{i}^{\left(a\right)}$ of the three weights as a function of time, compare Figure 1, along the corresponding rods**. The modes and parameters are identical to the ones presented in Figure 4. Time is measured in units of 2*s*.

It is evident that the body plan of the robot examined here tends to produce meandering motion pattern. T1 and T2 are sun- and star-like movements with small (T1) and large (T2) processing angles (compare Figure 4; “T” stands for torus in phase space). There is, in addition, a (nearly pure) circular motion, C1, and three types of forward snake-like meandering motion patters, S1, S2, and S3. From these S3 partly overlaps with itself. These modes are characterized by distinct motion patterns of the three weights, as shown in Figure 5, as measured by their positions along their respective rods. The differences between the distinct modes are in part qualitative, in terms of the time sequences in which the three neurons are subsequently active, and in part only quantitative. The difference between T1 and S1 is, in this respect, that the up-times of the two active neurons are symmetric for S1, but not for T1. A spontaneous symmetry breaking can be furthermore observed in case of T1, S1, S2, S3, for which two weights always have alternating dynamics, the third one showing a qualitatively different behavior. In contrast to that, the time-series of the C1 and T2 modes reveals the symmetrical but phase shifted oscillation of the three weights. Note that the positions of the weights may overshoot the interval [−*pR, pR*] for the target positions $x_{i}^{\left(t\right)}$, both due to inertia and due to the additional gravitational pull. Motion patterns similar to the ones shown in Figure 4 have been observed in a self-organized two-wheeled robot in the frozen mode (Der and Martius, 2013).

### 3.2. Chaotic modes allowing for explorative behavior

The dynamics of the robot takes place in a phase space combining the internal variables, of both body and controller, with the ones of the environment. The stability regions of the individual limit cycles presented in Figure 3 will therefore be bounded, generically, by a suitable bifurcation, such as a supercritical Hopf bifurcation or a fold bifurcation of limit cycles (Gros, 2015; Sándor et al., 2015). Alternatively, a transition to chaos may occur. It is on the other side also possible that chaotic attractors emerge from previously unstable manifolds and that the stability region of chaotic and stable manifolds overlap.

**Figure 6:**
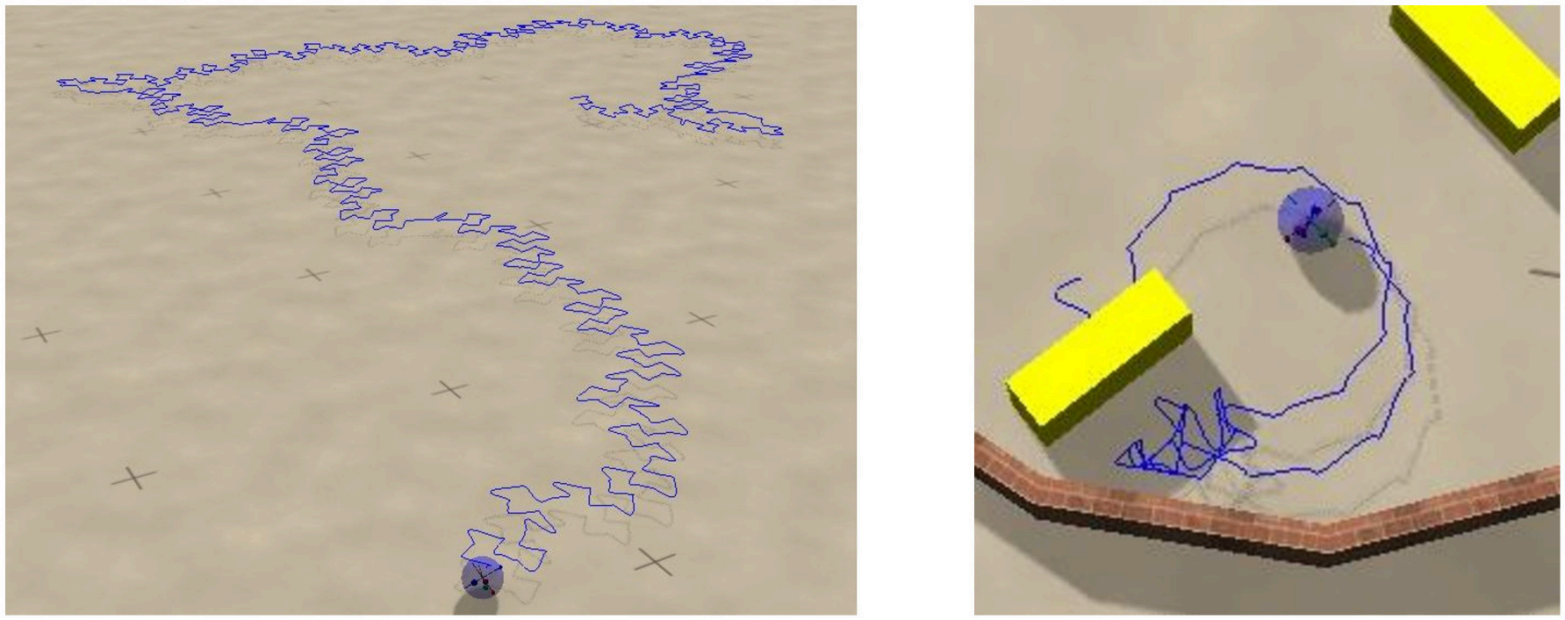
**Screenshots of the sphere robot in a chaotic mode; *U*_*max*_ = 1 and (*w*_0_, *z*_0_) = (210, 400)**. The blue lines retrace the past trajectory. The short-time motion of the robot is close to the one of the S2 mode, which is here an unstable attractor (compare Figure 4). **Left**: In open space. **Right**: In a closed environment allowing for the interaction with movable objects (yellow blocks). The circular sections correspond to unstable C1 limit cycles. A close-up to the dynamics and a longer simulation in the maze can be seen in Supplementary Videos 2, 3 respectively.

Close to a chaotic phase long transients may occur, which makes it difficult to study systematically the exact extend of the chaotic region. In Figure 3 we have indicated however a few representative combinations of parameters, for which stable chaos is observed both in the limit of long simulations times and for a wide range of stepsizes of the ODE simulator. No regular motion patterns can be observed in the screenshots presented in Figure 6. We have also evaluated the long-time behavior of the square of the covered real-space distance,

(5) $$d^{2}(\tau )\;=\;{\langle {\left(x(t+\tau )-x(t)\right)}^{2}\rangle }_{t}\;.$$

We found diffusive transport $d\sim \sqrt{\tau }$ for the chaotic mode and ballistic transport *d* ~ τ for the forward meandering modes S1, S2, and S3. Both as expected.

It has been observed, that chaotic locomotion of an embodied system may be considered as a basic explorative behavior, both of the environment and of the own motor pattern (Steingrube et al., 2010; Shim and Husbands, 2012). As a test of this hypothesis we have set our three-rod robot into a restricted playground containing movable objects in the form of blocks, which can be pushed, to a certain extend, over the ground. A screenshot is presented in Figure 6. One can observe, that the robot stays for a while close to the object, bumping around, and retracting in part a trajectory having a shape similar to the one generated by a C1 limit cycle. This is possible, as the set of parameters (*w*_0_, *z*_0_) = (210, 400) considered is located close to but outside the C1-stability region. The C1 limit cycle is hence only weakly unstable in the chaotic phase. The active exploration of the environment, occurring here when bumping into obstacles, gives the robot hence access to otherwise unstable locomotion options. The overall behavior may be interpreted alternatively in terms of non-representational sensorimotor knowledge (Buhrmann and Di Paolo, 2014). For a longer simulation see the Supplementary Videos.

In the movie presented in the Supplementary Material one can observe, furthermore, that the robot is pushing the blocks around in a seemingly “playful” manner (see Supplementary Video 3). A remarkable behavior, in our view, considering that the sphere robot disposes of a mere total of three controlling neurons. We note, that this complex behavior results from the interplay of the autonomous dynamics, as resulting from the inter-neural short-term synaptic plasticity, with environmental feedback.

### 3.3. Embodiment shaping the intrinsic dynamics

**Figure 7:**
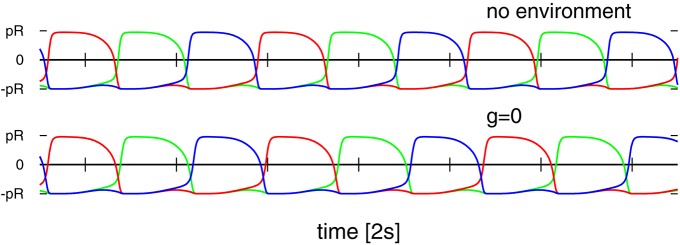
**Time series of the target positions $x_{i}^{\left(t\right)}$ for *U*_*max*_ = 1 and (*w*_0_, *z*_0_) = (190, 600), which correspond to the C1 mode shown in Figures 4, 5**. **Top**: For a numerical simulation of the isolated network obtained when setting $x_{i}^{\left(a\right)}=x_{i}^{\left(t\right)}$ in Equation (2). **Bottom**: For the 3-rod robot suspended in air (with the gravity constant *g* set to zero). Note that both time-series are very similar but not identical.

One can consider the controlling 3-neuron network in isolation by identifying the sensory reading $x_{i}^{\left(a\right)}$ for the actual position of the weight along the rod with the respective target position $x_{i}^{\left(t\right)}$, viz by setting $x_{i}^{\left(a\right)}=x_{i}^{\left(t\right)}$ in Equation (2). The resulting network contains a self-excitatory coupling *w*_0_ together with all-to-all inhibition with a bare synaptic strength *z*_0_. The short-term synaptic plasticity then induces an autonomous activity, as illustrated in Figure 7, which is topologically equivalent to the C1 mode. This equivalence becomes even more pronounced when suspending the robot in air, which can be achieved in turn by simply removing gravity from the physics simulation (bottom time-series in Figure 7). One can hence consider the C1 mode as the driver for the observed physical motion.

The isolated 3-neuron network has, however, only a single stable limit cycle. Numerically integrating the isolated network for parameters settings (*w*_0_, *z*_0_) corresponding to the six modes of Figure 5, as well as for chaotic states, we find always an identical sequential activation of the three neurons illustrated in Figure 7, with only slight changes in the overall shape. It is hence clear, that the other modes T1, T2, S1, S2, and S3, as well as the chaotic behavior, do result from the closed-loop feedback of the environment. The interaction of the environment with the intrinsic dynamics then results in the emergence of alternative types of locomotion.

### 3.4. Stability with respect to noise

**Figure 8:**
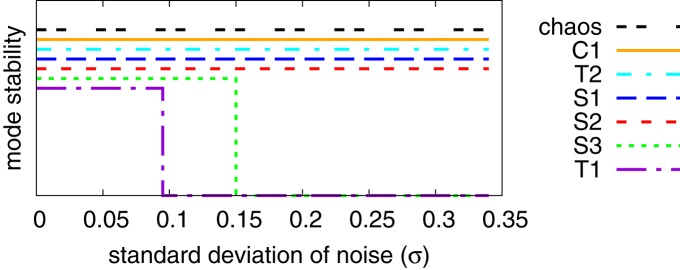
**Stability analysis of the modes found for *U*_*max*_ = 1, compare Figure 3, against a noise term Δ*x* in the sensory reading, defined by $x_{i}^{\left(a\right)}\to x_{i}^{\left(a\right)}\left(1+\Delta x\right)$**. Finite and zero values along the *y*-axis indicate stability and instability (the displacements along the *y*-axis are only for avoiding overcrowding). The noise Δ*x* is normal-distributed with standard deviation σ. Once T1 and S3 become unstable, when adiabatically increasing the noise level, their respective basins of attraction merge with the attracting regions of C1 and S1.

We present in Figure 8 an analysis of the stability of the various modes found, with respect to noise in the sensory readings, where the level of the noise is given by the relative standard deviation σ of the sensory readings $x_{i}^{\left(a\right)}$. Comparing with the phase diagram, as presented in Figure 3, one notices that first modes to disappear, T1 and S3, are the ones with small stability regions in the phase diagram. Ramping up the noise level the T1 and S3 modes turn respectively, above their corresponding critical noise levels, into C1 and S1 modes. The other modes, including the chaotic phase, are in contrast very stable with respect to noise.

### 3.5. Autonomous mode switching

**Figure 9:**
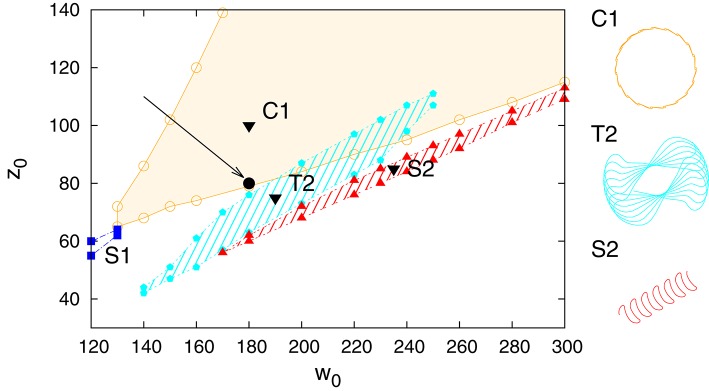
**The phase diagram obtained when using *U*_*max*_ = 4 in the STSP rules (Equation 2.1), with the naming of the modes corresponding to the ones used for the *U*_*max*_ = 1 phase diagram presented in Figure 3**. At (*w*_0_, *z*_0_) = (180, 80) there is chaotic state, as indicated by the arrow, coexisting with the C1 mode. The extent of the chaotic phase has not been examined in depth. On the right the traces are shown for the three dominant modes C1, T2, and S2.

We present in Figure 9 the phase diagram obtained when using *U*_*max*_ = 4 for the maximal Ca-level entering Equation (2.1). Within the range of (*w*_0_, *z*_0_) scanned we find four out of the six modes observed for *U*_*max*_ = 1 (compare Figure 3). The range of inhibitory weights *z*_0_ for which stable locomotion is found is rescaled down, in addition, with respect to the *U*_*max*_ = 1 case. Interestingly we found a chaotic state at (180, 80) which lies just inside the stability region of the C1 mode.

**Figure 10:**
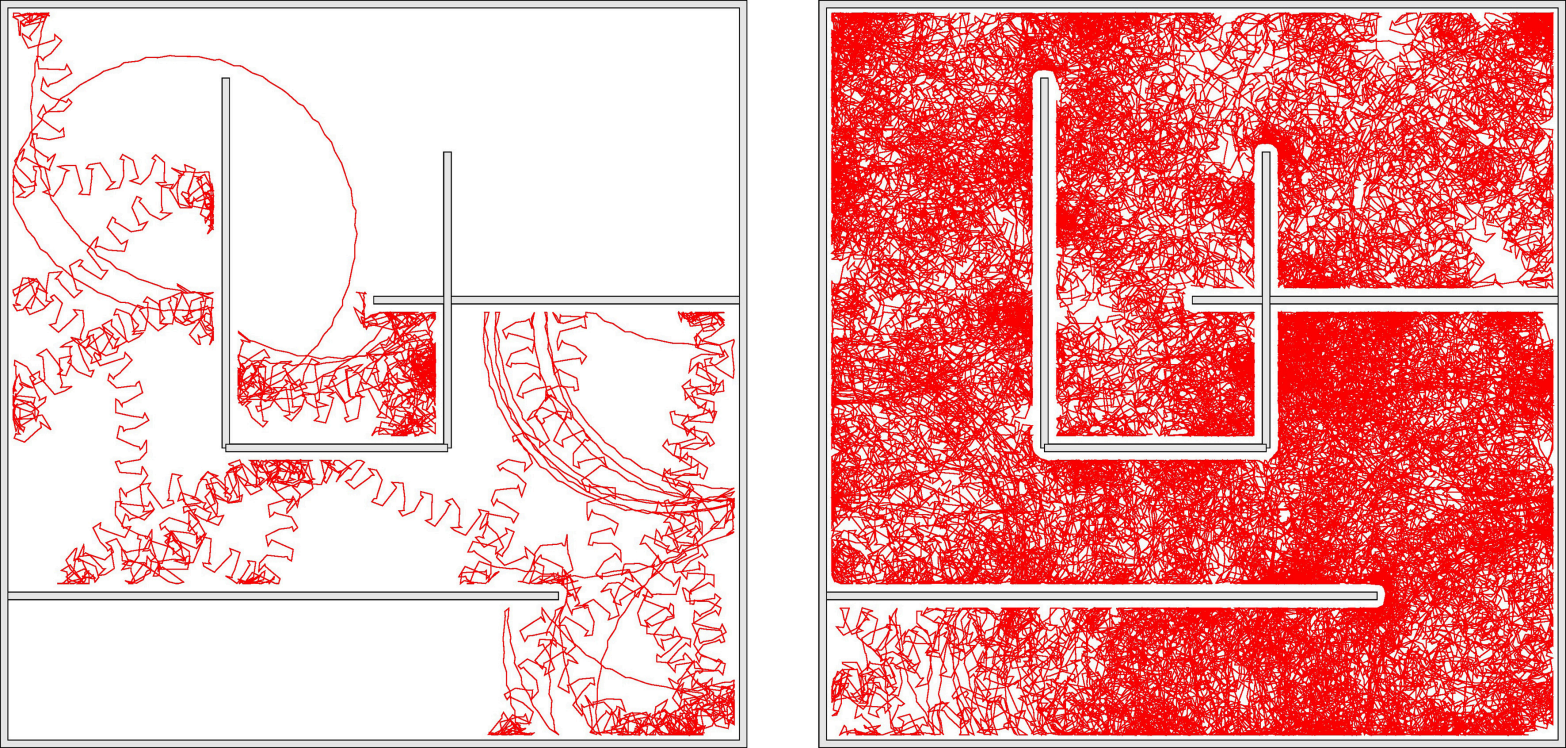
**The trace of the robot in a maze for a simulation time of 83 (left) and 1000 (right) min, respectively**. The robot may remain stuck occasionally in corners, but not forever. The parameters are *U*_*max*_ = 4 and (*w*_0_, *z*_0_) = (180, 80), corresponding to the chaotic mode indicated by the arrow in Figure 9. Bumping against the wall the robot sometimes turns up in the C1 mode, which is a coexisting stable limit cycle. The radius of the C1 mode is however, in this case, so large, that it does not fit as a whole into the maze. Also note that the chaotic mode is locally akin to the here unstable S2 mode, and that it changes the overall direction only on a relatively large scale.

We did let the robot evolve within the borders of a simple maze, as shown in Figure 10 and Supplementary Video 4. Most of the time the robot is in the chaotic state, which is the dominant mode for the parameters used, namely (*w*_0_, *z*_0_) = (180, 80) and *U*_*max*_ = 4. Intermittently, after colliding with a wall, the robot switches to the coexisting C1 mode. The radius of the stable C1 limit cycle in real-world coordinates is however so large, for (*w*_0_, *z*_0_) = (180, 80), that it does not fit into the maze. The robot hence continues exploring. We have obtained similar results when using a *U*_*max*_ = 1 chaotic mode.

**Figure 11:**
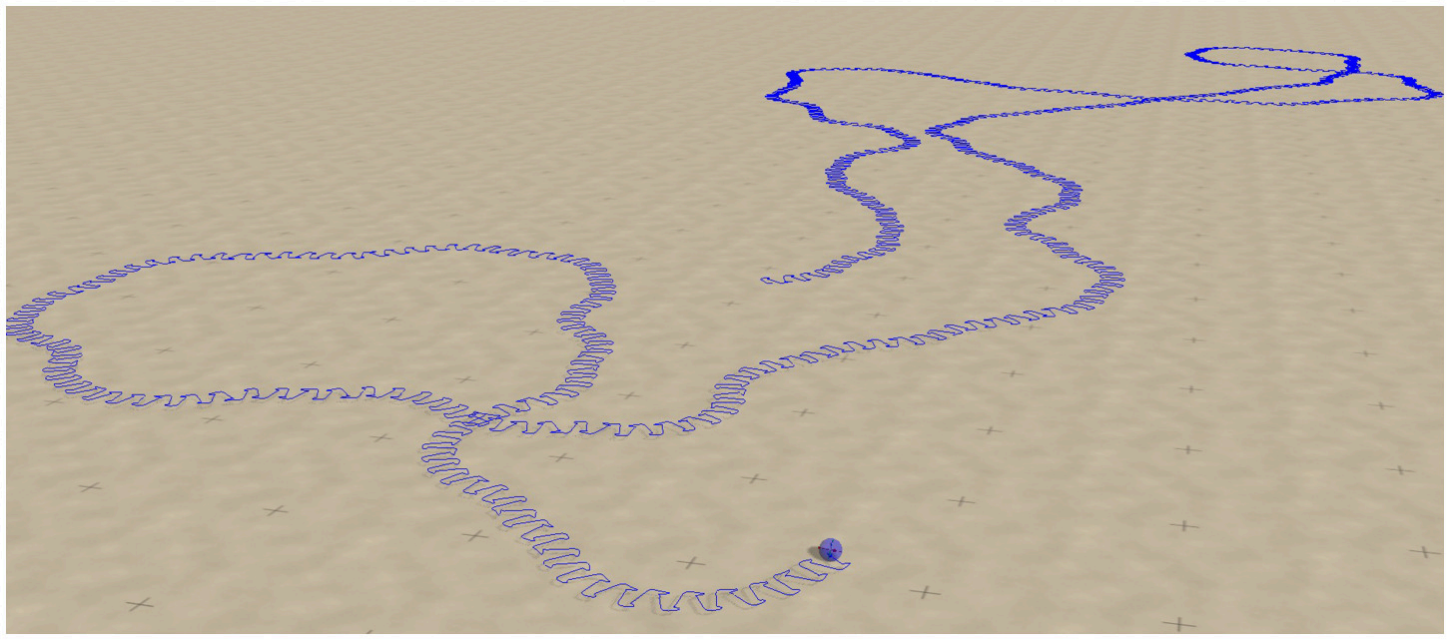
**Screenshot of the sphere robot in a chaotic mode for *U*_*max*_ = 4 and (*w*_0_, *z*_0_) = (180, 80), indicated by the arrow in Figure 9**. The blue line retraces the past trajectory. Note that the chaotic wandering is substantially smoother than the one observed for the *U*_*max*_ = 1 case (compare Figure 6).

A screenshot of a trajectory in open space is presented in Figure 11. One notices, that the *U*_*max*_ = 4 and (*w*_0_, *z*_0_) = (180, 80) chaotic mode wanders around aimlessly in much smother manner, than the *U*_*max*_ = 1 chaotic mode shown in Figure 6. This is the result of topologically different attractor structures, as seen in the phase space of internal variables (see the Supplementary Materials). Different types of chaos are indeed known to exist (Wernecke et al., 2016).

The autonomous mode switching observed for the regular motion primitives can also be seen in Supplementary Video 1. For a detailed discussion of the possible switching scenarios see the Supplementary Materials.

### 3.6. Switching between degenerate unstable limit cycles

**Figure 12:**
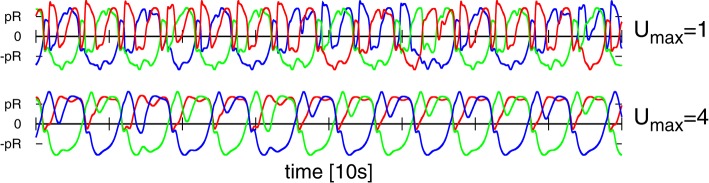
**As a function of time the positions of the three weights, compare Figure 1, along the corresponding rods**. **Top**: For the *U*_*max*_ = 1 chaotic mode with (*w*_0_, *z*_0_) = (210, 400) shown in Figure 6. **Bottom**: For the *U*_*max*_ = 4 chaotic mode (*w*_0_, *z*_0_) = (180, 80) shown in Figure 11. Both modes are locally akin to an S2 mode, albeit with substantial fluctuations (e.g., compare the bottom curvatures of the green line for *U*_*max*_ = 4, see also Figure 5). Note that phase slips do occur for the case of *U*_*max*_ = 1, but not for *U*_*max*_ = 4.

In Figure 12 we compare for the two chaotic modes, realized for *U*_*max*_ = 1 and for *U*_*max*_ = 4 respectively, the time series for the positions of the weights along the rods. One observes, that the movements of the weight is qualitatively similar, on short time scales, to an S2 mode (compare Figure 5, see also Supplementary Video 3). It is interesting, in this context, that the S2 mode has two types of degeneracies.

- Continuous. The S2 mode may propagate in any direction. There is hence a continuous manifold of attractors in the combined phase of controller, body and environment. Outside the actual region of stability this manifold contains either unstable limit cycles or limit cycle relicts (Gros, 2009).
- Discrete. There is a spontaneous symmetry breaking in the S2 mode, with two weights having identical but phase shifted movement patterns along their respective rods, which are qualitatively different to the trajectory of the third weight (see Figure 5).

For the *U*_*max*_ = 4 chaotic mode we did not observe discrete mode switching, in above sense, which however occurs frequently for the *U*_*max*_ = 1 mode (see Figure 12). The chaotic meandering observed for the *U*_*max*_ = 4 chaotic mode, as evident in Figure 11, is hence a consequence of a smooth diffusion of the angle of propagation on the manifold of unstable S2 limit cycles (or limit cycle relicts Linkerhand and Gros, 2013). In the phase space of the neural activity (as shown in Supplementary Figure 5), the trajectory corresponds to a chaotic phase diffusion along a limit cycle (Wernecke et al., 2016). This process is determinstic and not due to numerical errors, as we have checked by systematically reducting the stepsize used for the numerical integration. Noise is absent.

## 4. Conclusions

We have shown here, that a robot controlled by only a very limited number of neurons, three in our case, may show complex behavior which may be interpreted as explorative or playful. This is possible when locomotion results from self-organizing processes in the sensorimotor loop. The driving control dynamics, for which we have considered here short-term synaptic plasticity, then adapts itself seemingless to the physical requirements. No central controller is needed to detect an external object (Rai et al., 2014), or to switch direction when colliding with it. Stable and unstable limit cycles, together with chaotic attractors, arise in the phase space of internal (control and robot body) variables. These attractors form continua in the space of physical location and overall propagation direction, with the chaotic locomotion transitioning between unstable limit cycles. Transitions may either be between different types of regular locomotion, bounded circular or propagation meandering modes, or between the directions of unstable propagating limit cycles.

We note that the formation of a continuum of attractors is possible, whenever internal and external variables can be separated, such that internal variables span an independent subset of the phase space of the dynamical system. Here, the position of the robot (on the ground plane, in the absence of obstacles) acts as an external variable, all the other variables being independent of it. The limit cycles and chaotic attractors, living in the subspace of internal variables, exist thus for all position vectors, generating a continuous degeneracy of locomotion modes. The interactions with other robots and obstacles then results in a transient breakdown of this degeneracy, which is restored instantaneously with the termination of physical contact. Within this context, higher order control mechanisms would correspond to an external-variable dependent feedback, shaping the attractors either intermittently or slowly (with respect to the internal dynamics), thus leading possibly to the emergence of transiently stable attractors.

Our result, that the three-rod robot switches spontaneously between a continuous set of attractors, in the chaotic state, can be seen as a realization of chaotic wandering (Tsuda, 2001), which has been argued in turn to occur in the brain in the form of self-organized instabilities (Friston et al., 2012), viz as transient-state dynamics (Gros, 2007). There is furthermore a close relation to the concept of attractor metadynamics (Gros et al., 2014), which denotes the either induced or spontaneous switching between attracting sets.

The here simulated robot is furthermore compliant both on the level of control and actuators, showing a highly flexible response. The actuators are implemented by specifying a target position for a limb, here a moving weight on a rod. The force acting on the weight then results from the interplay between the internal driving, provided by a damped spring (between the actual and the target position), with the physical restoring forces acting on the weights, which in turn depend on the body dynamics determined by the interaction with the ground, obstacles and other robots (Floreano et al., 2014).

The isolated controlling network (realized in the limit of infinitely strong actuators) can be interpreted in addition as a central pattern generator (Steingrube et al., 2010), having a single intrinsic limit-cycle attractor. The open-loop control incorporates however the feedback of the environment through the induced forces. We find here, that the resulting embodiment (Cangelosi et al., 2015) does morph the driving dynamics of the central pattern generator not only quantitatively, but also qualitatively, giving rise to a vast array of modes which differ in part topologically from the dynamics of the underlying central pattern generator. We believe that this dynamical systems approach of the locomotion of simple robots has not been fully exploited yet, having many interesting features and applications in store for the field of neurorobotics.

## Author contributions

The experiments were conceived and designed by CG, BS, and LM, performed mainly by LM with BS adding some data. The data was analyzed by CG, BS, and LM, most of the plots produced by LM. The manuscript was mostly written by CG, with BS adding some paragraphs and revising it with LM.

### Conflict of interest statement

The authors declare that the research was conducted in the absence of any commercial or financial relationships that could be construed as a potential conflict of interest.
