# Supplementary material for: Closed-loop Robots Driven by Short-Term Synaptic Plasticity: Emergent Explorative vs. Limit-Cycle Locomotion
Source: Front Neurorobot. 2016 Oct 18;10:12. doi: 10.3389/fnbot.2016.00012 (PMC5067527; doi:10.3389/fnbot.2016.00012)
Supplement: Supplementary file 5 [file Presentation1.pdf]

Supplementary Material: Closed-loop robots driven by short-term synaptic plasticity: Emergent explorative vs. limit-cycle locomotion

Laura Martin [1], Bulcsú Sándor [1,*] and Claudius Gros [1]
*Correspondence:
Bulcsú Sándor
sandor[.]itp.uni-frankfurt.de

1 SUPPLEMENTARY VIDEOS

Here we provide supplementary videos to the different dynamical behaviors discussed in the main paper. The simulations can be reproduced by using the LpzRobots simulation software, available on http://robot.informatik.uni-leipzig.de/software
https://github.com/georgmartius/lpzrobots.

1.1 Regular motion patterns

The different locomotion modes found for $U_{max} = 1$ (see Fig. 3) are compared in Suppl. Video 1. Six robots are created with the same settings, except for the (w_0, z_0) parameter pairs, corresponding to the examples presented in Fig. 4: T1: $(280, 650)$, T2: $(230, 415)$, C1: $(190, 600)$, S1: $(250, 530)$, S2: $(240, 380)$, S3: $(220, 470)$. As result of locomotion the robots interact. Following the individual trajectories, among other interesting phenomena, autonomous mode switching is observed after collisions:

- The magenta robot displays the T1 mode, which coexists with the S1 and C1 motion patterns. Bumping into the red robot, first the center of the star-shaped trajectory is shifted. After the second collision it switches to the C1 mode, precessing transiently to the left, while as a result of a third collision it sets into the rightwards circulating mode.
- The light-blue (cyan) colored robot stays in T2 mode even after the collision, since for these parameters (see Fig. 4) other stable motion pattern does not exist.
- The same holds for the C1 mode as well, demonstrated by the orange robot. Note that the radii of the circular patterns generated by the orange and the magenta robots differ significantly, an effect of the significantly different w_0 parameters.
- The S1 mode is visualized by the dark-blue spherical robot. The collision induces an autonomous switch to C1.
- S2 mode is essential for our results, being a singleton locomotive mode in its parameter domain. Hence interactions with other robots or walls can only change the direction of translation. In Suppl. Video 1 one can observe seven such collisions.
- The green robot, originally started in S3 mode, changes to S1 on an early impact.

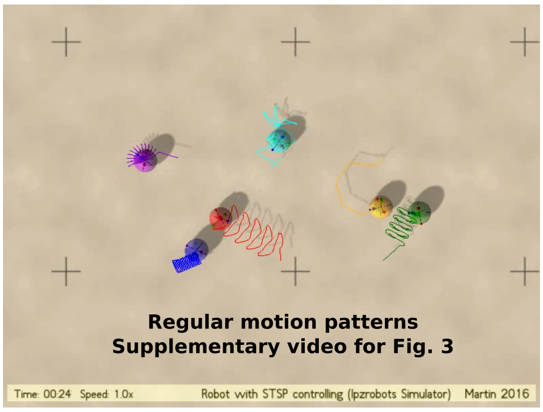

Supplementary Video 1. Examples of regular motions patterns T1, T2, C1, S1, S2, S3, generated by identical robots, using $U_{max} = 1$. The (w_0, z_0) parameter pairs are set for each robots separately, corresponding to the values used for the close-up of the trajectories in Fig. 4 (having initially the same order and color coding). When several modes coexist for the same parameters, robots interacting with each other can change their behavior, choosing an other working mode. See for instance the green robot, changing from S3 to S1 after colliding with the yellow one. See: Video 1.avi.

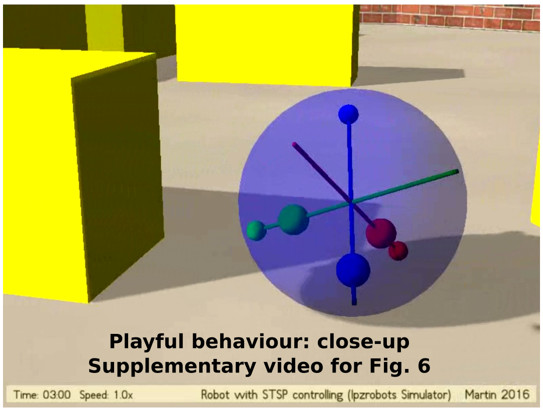

Supplementary Video 2. Close-up showing the dynamics of the robot in the chaotic mode, using $U_{max} = 1$ and $(w_0, z_0) = (210, 400)$. The smaller balls fixed to the end of the rods are guides to the eyes, indicating the negative direction along the axes, without any physical influence on the dynamics. The obstacles, having similar masses to the mass of the robot, are pushed apart in different directions. See: Video 2.avi.

1.2 Playful behavior

The playful behavior of the spherical robot is shown in Suppl. Video 2 and 3, for the parameter settings used for Fig. 6, $U_{max} = 4$ and $(w_0, z_0) = (210, 400)$. Autonomous mode switching can be observed between the coexisting chaotic meandering and C1 modes, pushing objects, of masses similar to its own body mass, in a playful manner.

1.3 Explorative behavior

In Suppl. Video 4 the two types of chaotic explorations are compared in a maze, also shown in Fig. 10, using $U_{max} = 1$ and $U_{max} = 4$. Additional stable modes also arise in environments different from the bare plane ground (see the locomotion along the walls).

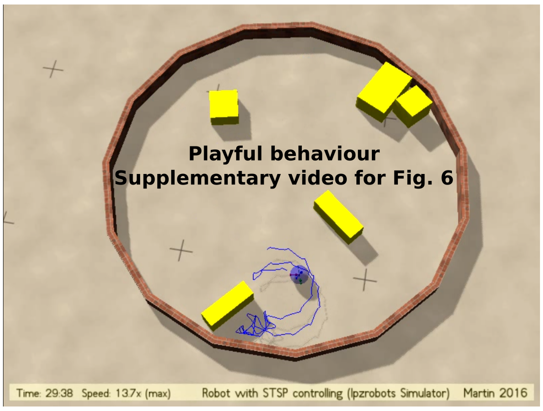

Supplementary Video 3. The playful behavior of the robot in the closed playground, viewed from the top. The parameters are set as for Suppl. Video 2, corresponding to the chaotic mode. The blue trajectory, plotted on the ground, traces the C1 locomotion pattern occasionally out, which is unstable for these settings (as discussed in the main text of the paper). See: Video 3.avi.

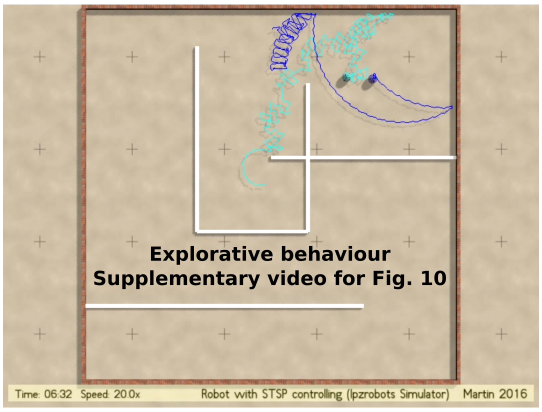

Supplementary Video 4. Two robots showing explorative chaotic behavior, placed in a maze with fixed walls, using $U_{max} = 1$, $(w_0, z_0) = (210, 400)$ for the cyan, and $U_{max} = 4$, $(w_0, z_0) = (180, 80)$ for the dark-blue colored robot. Note that the robots also interact occasionally. See: Video 4.avi.

2 CHAOTIC MODES

To examine the different modes from a dynamical systems point of view, one can consider the corresponding attractors in the subspace of internal variables (see also (Sándor et al., 2015)). The chaotic attractor corresponding to the explorative behavior with abrupt change of directions, found for $U_{max} = 1$, has a densely filling structure, as shown in Suppl. Fig. 5. For $U_{max} = 4$, with the angle of propagation only smoothly diffusing, however, the topology resembles closed braids, a sign of partial predictability, typically found close to period doubling bifurcations (Wernecke et al., 2016). As a result of the attractor's topology the discrete mode switching can never occur for $U_{max} = 4$ (see Fig. 12).

Note that for $U_{max} = 4$ there are totally three chaotic attractors in the phase space of internal variables, which are ultimately generating the same type of explorative behavior, being related by symmetry operations (corresponding to the permutation of colors in Fig. 12). Hence, in this case discrete mode switching (between these attractors), similar to the S2 case, can be achieved by adding external noise.

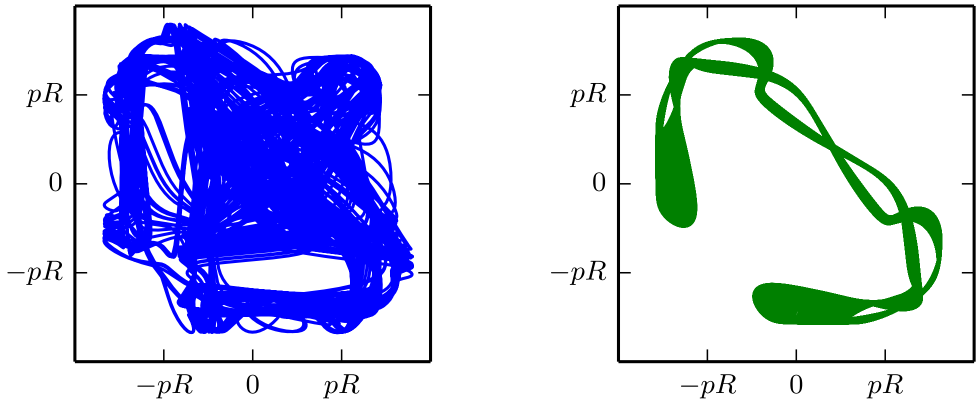

Supplementary Figure 5. Projection of the chaotic attractors to the phase plane of actual positions $(x_2^{(a)}, x_3^{(a)})$, using $(w_0, z_0) = (210, 400)$ with $U_{max} = 1$ on the left, and $(w_0, z_0) = (180, 80)$ with $U_{max} = 4$ on the right (only one shown of the three).

REFERENCES

Sándor, B., Jahn, T., Martin, L., and Gros, C. (2015). The sensorimotor loop as a dynamical system: How regular motion primitives may emerge from self-organized limit cycles. *Frontiers in Robotics and AI* 2, 31

Wernecke, H., Sándor, B., and Gros, C. (2016). Partially predictable chaos. *arXiv preprint arXiv:1605.05616*
